# Adaptive laboratory evolution in *S. cerevisiae* highlights role of transcription factors in fungal xenobiotic resistance

**DOI:** 10.1038/s42003-022-03076-7

**Published:** 2022-02-11

**Authors:** Sabine Ottilie, Madeline R. Luth, Erich Hellemann, Gregory M. Goldgof, Eddy Vigil, Prianka Kumar, Andrea L. Cheung, Miranda Song, Karla P. Godinez-Macias, Krypton Carolino, Jennifer Yang, Gisel Lopez, Matthew Abraham, Maureen Tarsio, Emmanuelle LeBlanc, Luke Whitesell, Jake Schenken, Felicia Gunawan, Reysha Patel, Joshua Smith, Melissa S. Love, Roy M. Williams, Case W. McNamara, William H. Gerwick, Trey Ideker, Yo Suzuki, Dyann F. Wirth, Amanda K. Lukens, Patricia M. Kane, Leah E. Cowen, Jacob D. Durrant, Elizabeth A. Winzeler

**Affiliations:** 1 Department of Pediatrics, University of California, San Diego, Gilman Dr, La Jolla, CA 92093 USA; 2 Department of Biological Sciences, University of Pittsburgh, 4249 Fifth Avenue, Pittsburgh, PA 15260 USA; 3 Department of Biochemistry and Molecular Biology, SUNY Upstate Medical University, Syracuse, New York, NY 13210 USA; 4 Department of Molecular Genetics, University of Toronto, Toronto, ON M5G 1M1 Canada; 5 Calibr, a division of The Scripps Research Institutes, La Jolla, CA 92037 USA; 6 Center for Marine Biotechnology and Biomedicine, Scripps Institution of Oceanography, La Jolla, CA 92037 USA; 7 Department of Medicine, University of California San Diego, La Jolla, CA USA; 8 Department of Synthetic Biology and Bioenergy, J. Craig Venter Institute, La Jolla, CA 92037 USA; 9 Department of Immunology and Infectious Diseases, Harvard T.H. Chan School of Public Health, Boston, MA USA; 10 Infectious Disease and Microbiome Program, Broad Institute, Cambridge, MA 02142 USA; 11 Present Address: Aspen Neuroscience, San Diego, CA 92121 USA

**Keywords:** Evolutionary biology, Target identification

## Abstract

In vitro evolution and whole genome analysis were used to comprehensively identify the genetic determinants of chemical resistance in *Saccharomyces cerevisiae*. Sequence analysis identified many genes contributing to the resistance phenotype as well as numerous amino acids in potential targets that may play a role in compound binding. Our work shows that compound-target pairs can be conserved across multiple species. The set of 25 most frequently mutated genes was enriched for transcription factors, and for almost 25 percent of the compounds, resistance was mediated by one of 100 independently derived, gain-of-function SNVs found in a 170 amino acid domain in the two Zn_2_C_6_ transcription factors *YRR1* and *YRM1* (*p* < 1 × 10^−100^). This remarkable enrichment for transcription factors as drug resistance genes highlights their important role in the evolution of antifungal xenobiotic resistance and underscores the challenge to develop antifungal treatments that maintain potency.

## Abstract (web summary)

Ottilie et al. employ an experimental evolution approach to investigate the role of transcription factors in yeast chemical resistance. Most emergent mutations in resistant strains were enriched in transcription factor coding genes, highlighting their importance in drug resistance.

## Introduction

Experimental evolution is an important method for studying evolutionary processes and is especially amenable to microorganisms given their short generation times, large population sizes, and ability to cryopreserve at distinct timepoints^1^. The longest-running and largest-scale laboratory evolution experiment to date utilize *E. coli*^2–4^, although the approach has been used successfully in additional microbes, including the eukaryote *S. cerevisiae*^5–7^. While many laboratory evolution experiments focus on changing nutrient availability or culturing conditions, another application of in vitro evolution involves studying an organism’s evolutionary response to drug pressure. For example, many antibiotic resistance mechanisms have been identified and characterized through this method^8–10^. A key challenge lies in identifying functional variants that contribute to the evolved phenotypes given the sheer number of mutation events; a recent sequencing study of 1011 yeast isolates identified 1,625,809 SNVs^11^.

Systematic functional genomic studies are also used to understand drug-target interactions, but most rely on strain libraries in which the entire coding region is modified. For example, a set of homozygous and heterozygous knockout yeast strains was constructed which bear deletions in all genes in the genome^12,13^. This set has been used to repeatedly and systematically identify knockout/knockdown lines that show sensitivity or resistance to a wide variety of different compounds^14^ and remains important^15^. CRISPR-based, genome-wide knockout and knockdown studies and novel genome editing systems like base editors are also used in many organisms to identify drug targets^16–19^ or study processes such as the emergence of cancer drug resistance. A key limitation of such studies is that they will miss gain-of-function SNVs, which often drive natural adaptive evolution^20^. Some examples of gain-of-function antibiotic resistance mechanisms include mutations that activate global transcription factors^21^ and mutations within the promoter region of resistance genes that result in their hyperproduction^22–24^.

Here, we perform in vitro evolution and whole-genome analysis (IVIEWGA) in *S. cerevisiae* to delineate drug-target interactions with a large set of compounds and in the process, extensively characterize key features of the yeast resistome. Whole-genome sequencing of 355 evolved, compound-resistant clones showed only a few new coding variants per clone and that statistical approaches can be used to readily identify variants that modify phenotypes. As a proof-of-concept, we confirmed targets that were previously reported and identified additional resistance-conferring mutations for molecules with unknown mechanisms of action (MOA). We also observed enrichment for gain-of-function variants that affect transcription and confer resistance to multiple compounds.

## Results

### Building a library of compounds that are active against a drug-sensitive yeast

To understand how yeast evolves to evade the action of small molecules, identify genes contributing to resistance to uncharacterized small molecules, and hone in on domains that might contribute to compound-target interaction, we tested a collection of molecules for activity against *S. cerevisiae*. Since wild-type *S. cerevisiae* (strain S288C) requires higher compound concentrations to inhibit growth due to an abundance of drug export pumps (ABC transporters), in vitro selection would be impossible for many compounds. Therefore, to find compounds with activity against yeast at physiologically relevant concentrations, we used a modified *S. cerevisiae* strain, termed the “ABC_16_-Green Monster” (GM), in which 16 ABC transporters have been replaced with a green fluorescent protein (GFP)^25^. This has proven an excellent platform for in vitro drug selection and target identification experiments because of lower compound requirements^26–30^.

**Fig. 1:**
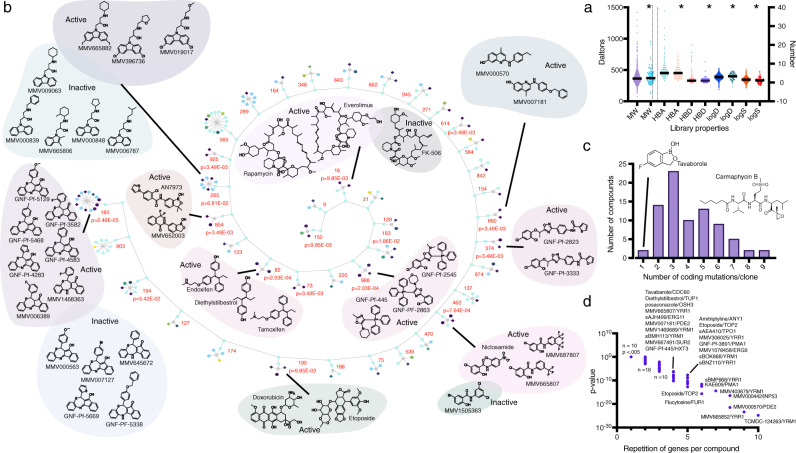
Compound summary. **a** Lipinski properties. Lipinski’s properties of compounds used in this study were calculated using StarDrop version 6.6.4. Left *Y*-axis: MW molecular weight: Right *Y*-axis: HBD hydrogen bond donor, HBA hydrogen bond acceptor, logD, logS. * indicates 80 compounds that yielded resistant clones. **b** Maximum Common Substructure (MCS). Structure similarity clustering analysis for 80 compounds yielding resistant clones and larger library of 1600, using Tanimoto as the similarity metric. The diagram shows 41 clusters sharing an MCS from which at least one compound was selected for drug response (indicated by diamonds). Circles represent compounds that were not selected, or inactive. The strength of cytotoxicity against the *S. cerevisiae* GM strain of tested compounds is indicated by the node’s color intensity from purple (higher potency) to yellow (lower potency). Probability values were calculated using the hypergeometric mean function showing that enrichment for clusters was greater than expected by chance. Compounds from clusters with a *p*-value of less than 0.05 and which had multiple members active against GM are shown. **c** Coding region mutations for selected compounds. Histogram showing the distribution of the number of coding mutations (e.g., missense, start-lost) per clone for the set of 80 compounds used in selections. **d** Gene enrichment for selected compounds. The *p*-value is the probability of repeatedly discovering the same gene for a given compound, calculated using Bonferroni-corrected hypergeometric mean function as described in Methods. Compound/gene pairs for *n* = 1, 2, and 3 can be obtained from Supplementary Data 4.

Specifically, we evaluated compound libraries comprised of (1) drugs approved for human use with characterized MOAs, (2) well-known tool compounds, and (3) compounds from open-source libraries with demonstrated activity against eukaryotic pathogens, viruses, or tuberculosis^31,32^ (Supplementary Data 1). Commercially available compounds were tested in dose-response, while other libraries were initially tested at a single point concentration (in biological duplicates) of up to 150 µM, the maximum concentration possible to avoid in-assay DMSO toxicity. Compounds that showed at least 70% growth inhibition were subsequently tested in dose-response. Overall, the compounds of the assembled collection had drug-like physiochemical properties in terms of molecular weight and the number of hydrogen bond donors and acceptors (Fig. 1a). Maximum Common Substructure (MCS) clustering identified 307 clusters with a Tanimoto similarity coefficient of 0.64 (Fig. 1b, Supplementary Data 1). Cluster enrichment was observed at rates greater than expected by chance. For example, there were 12 members of a benzothiazepine family (cluster 185, Fig. 1b) of which six had an IC_50_ of less than 10 µM in the yeast model (*p* = 2.46 × 10^−5^).

### In vitro resistance evolution and whole-genome analysis link compound structures to phenotype

**Fig. 2:**
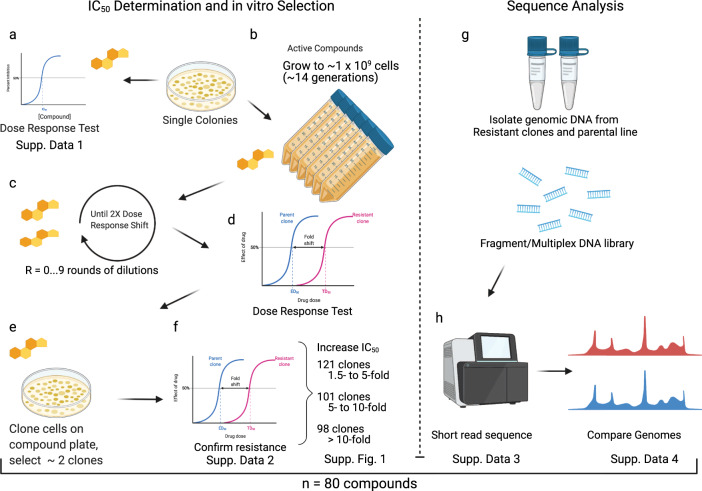
Generation of resistant yeast strains using a stepwise method of compound exposure. **a** To determine the degree of growth inhibition of small molecules, cultures derived from single colonies of the ABC_16_-Green Monster strain (GM) were exposed to various drugs and the IC_50_s determined. **b** For in vitro selections single colonies of GM were picked and grown to saturation in YPD media. 50 μl cells of a saturated culture (OD_600_ = 1) were inoculated into 50 mL tubes containing 20 mL of YPD media with a small-molecule inhibitor and grown until saturation. The starting drug concentration was the pre-determined IC_50_. **c** Upon reaching saturation cultures were diluted (1:400) into fresh media with increasing drug concentrations. **d** Development of resistance was evaluated through regular IC_50_ determinations. **e** Once cultures showed at least a 2-fold shift in IC_50_ single clones were generated by plating an aliquot of the resistant strain onto compound-containing YPD plates. **f** Two independent clones were picked and the IC_50_ shift confirmed. IC_50_ values were calculated by subtracting OD_600_ nm values at time 0 h from time 18 h. Nonlinear regression on log([inhibitor]) vs. response with variable slope was performed using GraphPad Prism. Cycloheximide was used as a negative control. **g** DNA from clones deemed to be resistant (through a combination of fold shift of IC_50_ and *p*-value) was isolated and their whole-genome analyzed. **h** The genomes of the drug-naïve parents and the drug-resistant clones were compared and allele differences between these two clones were determined. Data from all in vitro evolutions was analyzed in great detail. To further validate the potential resistance of the identified mutations allelic replacement of these SNVs into the parental line through CRISPR-Cas9 was performed. Graphics created with Biorender.com under BioRender’s Academic License Terms.

Based on potency and compound availability, we selected 100 active compounds for follow-up in vitro evolution experiments to verify known MOAs, validate the use of this modified yeast strain in target deconvolution studies, and characterize crucial amino acids that disrupt compound binding when mutated. For each independent selection, ~500,000 cells derived from an ABC_16_-Green Monster single colony were grown to saturation (OD_600_ = 1.0–1.5) in 20 mL YPD media in the presence of sublethal compound concentrations equal to the previously determined IC_50_ (Fig. 2). After any of the independent cultures reached saturation (OD_600_ = 1.0–1.5), ~500,000 cells were transferred to a new 20 mL culture containing increasing concentration of the compound until resistance was observed as measured by an increase in IC_50_ compared to the parental strain. Although growth rates of individual clones in culture could be variable, each dilution series (1 × 10^9^ cells) was nevertheless grown to saturation (on average ~14 generations amounting to a selection time of 5–20 days before plating on solid media). Cultures were considered resistant if they (1) continued to grow at compound concentrations 2–3-fold above the IC_50_ value of the untreated culture, and (2) had at least a 1.5-fold shift in IC_50_ value compared to the drug-naïve parental line (Supplementary Data 2). After a variable number of resistance cycles (R average = 2.93, range = 1–9), these resistant cultures were plated on drug-containing plates with the compound concentration of at least 2-fold IC_50_ to isolate single clones. We picked two independent colonies from the drug-containing plates to verify the resistance phenotype before submitting DNA for whole-genome sequencing. We attempted up to 12 independent selections per compound and obtained 1–12 resistant clones. Using this strategy, we isolated 355 clones selected against 80 compounds. The IC_50_ values of the resistant clones increased 1.5–5-fold for 121 clones, 5–10-fold for 101 clones, and >10-fold for 98 resistant clones (Supplementary Data 2, Supplementary Fig. 1). Some selections were performed in a modified ABC_16_-Green Monster clone with *YRR1* deleted (see below). Culture contamination or poor compound availability accounted for most failed selections (20 compounds).

Next, resistant clones were sequenced to 55-fold average coverage (Supplementary Data 3) using a short-read methodology. To detect mutations that arose during compound selection, we designed a custom whole-genome analysis pipeline and filtering method (see Methods). In total, we discovered 1405 mutations (1286 SNVs and 119 INDELs) that met the filtering criteria, with an average of 3.96 mutations per clone (Supplementary Data 4). 1117 mutations occurred in coding regions while 288 mutations were intergenic, intronic, or splice region variants. 781 unique genes were mutated across the dataset. We typically observed between 1 and 8 coding mutations per evolved clone per compound (Fig. 1c) with some variation. For 53 of the 80 compounds, we observed statistically significant (*p* < 0.05) reproducibility with respect to genes that were mutated, with enrichment considerably over that expected by chance (Fig. 1d). For example, we obtained 13 independent TCMDC-124263-resistant clones with 52 mutations (38 coding), of which  10 were in a single gene, *YRM1*. Given that yeast has roughly 6000 genes, the Bonferroni-corrected probability of this enrichment by chance is 1.53 × 10^−25^. For this example, 9 of the 10 nucleotide changes in *YRM1* were clearly independent (Supplementary Data 4).

**Fig. 3:**
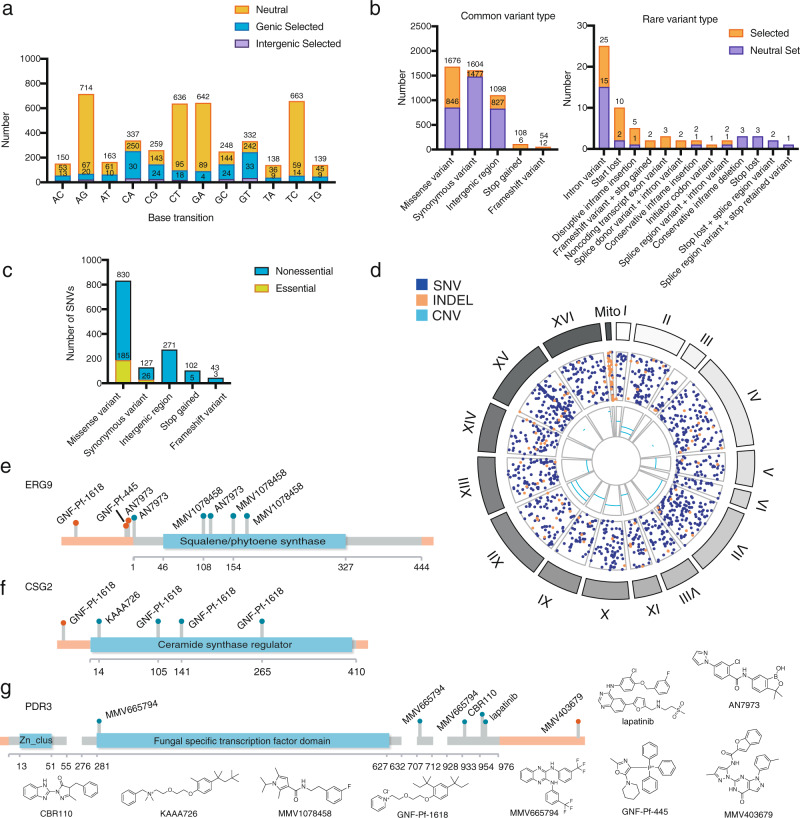
Mutations observed in yeast IVIEWGA experiments. **a** Base transition. Classification of mutation based on base transition type for 1286 SNV mutations obtained via compound selection and absence of compound selection^105^. **b** Classification of the most common mutation types in the dataset and their occurrence in essential vs. nonessential genes. Essentiality data were imported from the *Saccharomyces* Genome Deletion Project database and proportion of essential vs. nonessential genes that contained missense, synonymous, frameshift, and stop gained mutations were calculated. **c** Mutation type. Variant classes for 1405 mutations (INDELs and SNVs supplied from Supplementary Data 4) obtained via compound selection versus a mutation dataset from non-compound selection conditions^105^. **d** Circos plot. Circos plot of SNVs (blue), INDELs (orange), and CNVs (cyan) identified through resistance generation, generated with BioCircos R package^106^. **e**–**g** Intergenic mutations. Plot locating each coding region mutation onto the gene (gray) and intergenic mutation onto the chromosome (orange) based on the calculated distance. Mutations were mapped to their corresponding genomic location using *S. cerevisiae* S288C genome version R64-2-1. Intergenic mutations are located at no more than 500 bp away from the start/end of the gene.

To further assess how our evolved mutations were driving the observed resistance phenotypes, we considered the types of mutations that arose during selection and compared them to a published set of 3137 mutations in yeast strains grown long-term in the absence of compound selection^33^. We observed significantly different distributions in our compound-selected set than in the non-compound-selected set (χ^2^, *p* < 0.0001). For example, 39% of the nucleotide base transitions for 1286 SNVs in our compound-selected dataset were C to A or G to T, while for the 3137 transitions in the non-selected set, 40% were for A to G or T to C (Fig. 3a). Likewise, we observed a noteworthy difference in the coding changes. Among exonic selected mutations, 941 were nonsynonymous and 127 were synonymous. The estimated ratio of divergence at nonsynonymous and synonymous sites (dN/dS) was 2.62 across the dataset, indicating that drug treatment applied positive selection. In contrast, the neutral SNVs had a strong bias toward synonymous mutations (Fig. 3b). These data suggest that the observed mutations in our dataset are likely functional and could provide a selective advantage to the evolved clones.

We also assessed our large mutational dataset to identify broad insights into the functional impacts of different variant types. Synonymous and missense variants emerged in essential genes in ~20% of cases. This finding agrees with the literature, which suggests that only 20% of the yeast genome encodes essential genes^34^. By this same metric, mutation types with more disruptive impacts, such as premature stop codons and frameshift variants, deviate strikingly from the expected genome-wide value of 20%. These mutations occur in essential genes only 4.9% and 7.0% of the time, respectively (Fig. 3c).

Copy number variants (CNVs) were detected through a coverage-based algorithm using the output from GATK DiagnoseTargets to identify contiguous genic regions with increased read coverage relative to the parent. We observed 24 CNVs including apparent aneuploidy (11 times, occurring in 10 clones) and small, intrachromosomal amplification (13 times, occurring in 13 clones) (Supplementary Data 5). Altogether, we observed aneuploidy with 8 compounds, including BMS-983970, doxorubicin, etoposide, GNF-Pf-3582, GNF-Pf-4739, hygromycin B, CBR110, and wortmannin (Fig. 3d). This is perhaps not surprising given that aneuploidies arise in *S. cerevisiae* as a short-term stress response^35^. We observed an amplification on chromosome XVI that involved the bZIP transcription factor, *ARR1*, for clones resistant to GNF-Pf-1618 and GNF-Pf-2740, as well as with four strains resistant to MMV665794. The strains Wortmannin-13R3a and CBR113-7R4a, both had chromosome XV CNVs that involved the transcription factors *YRR1* and *YRM1* (discussed below).

### Resistance-conferring intergenic mutations are rare

Although intergenic mutations are frequently found in cells not subject to selection, mutations in promoters or 3’ UTRs could confer resistance by increasing or decreasing transcript levels. To assess examples where this might be the case, we mapped the 271 intergenic mutations identified across the sequencing dataset to their nearest-neighbor coding genes and other genomic features (Supplementary Data 6). This analysis showed little enrichment, although we did observe several repeated mutations located in the intergenic promoter regions of a few genes that showed significance in our enrichment analysis. For example, we discovered three mutations upstream of the ergosterol biosynthesis and azole resistance gene *ERG9*^36,37^ in addition to seven *ERG9* coding region mutations. One of the *ERG9* intergenic mutations falls in its putative promoter region and was also observed in selections with compound AN7973 (Fig. 3e). Four coding and one noncoding mutation upstream of the start codon in the endoplasmic reticulum membrane protein and caspofungin resistance protein^38^, *CSG2*, were also observed. All five mutations are associated with selections to compound GNF-Pf-1618 and its close analog, KAAA725 (Fig. 3f). We also identified five mutations in the coding region of *PDR3*^39^, a Zn_2_C_6_ transcriptional regulator of the multidrug efflux, and an additional mutation was found downstream of the open reading frame (Fig. 3g). These examples demonstrate that intergenic mutations should not be entirely dismissed when considering the effects of variant types on observed phenotypes.

### CRISPR/Cas9 validation shows that most genes identified more than once confer resistance, but singleton mutations may not

Because many mutations can co-occur and be non-adapative^40^, the presence of one SNV in a resistant clone is not proof that the specific mutation is resistance-conferring. To confirm that some of these mutations directly contribute to the resistance phenotype and rule out the possibility of them being merely passenger mutations, we used CRISPR/Cas9 technology to introduce 61 altered alleles from the evolved mutants back into the original (unevolved) ABC_16_-Green Monster strain. Mutations were chosen for validation based on the frequency of mutation and/or whether the gene product was implicated as a potential target for the compound based on literature searches. Successfully reverse-engineered strains were tested in liquid-growth assays using the same compounds from the corresponding IVIEWGA experiments. Using a combination of fold change and *p*-value in comparing the IC_50_ values of these edited clones to those of the parental ABC_16_-Green Monster strain, we verified that 45 genetic changes across 37 unique genes contributed to the observed resistance (Supplementary Data 7). Independent mutations that were repeatedly identified for a specific gene tended to have a high probability of confirming at least a partial resistance phenotype and these gene products were likely to be the direct target of the small molecule. The only exception was *RPO21*, a subunit of RNA polymerase, which was mutated four separate times with four compounds (two nonsynonymous and two synonymous mutations) but failed to confirm a resistance phenotype after CRISPR/Cas9 editing. However, it is known that mutations in *RPO21* result in transcriptional slippage, which may allow cells to better survive cytotoxic drugs that alter nucleotide pools^41^. For the 15 alleles that did not show a statistically significant IC_50_ shift (Supplementary Data 7), we noted that 11 of the resistant clones also carried additional resistance alleles in a highly represented gene such as *YRR1* or *YRM1* (Supplementary Data 4), two transcription factors involved in multidrug resistance.

### Using in vitro evolution for drug target and mechanism of action studies

**Fig. 4:**
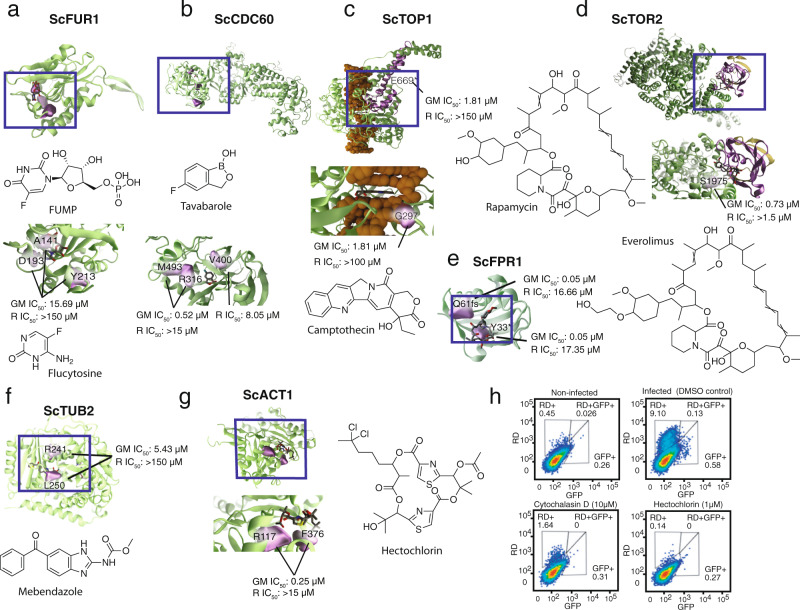
Resistance-conferring mutations in detail. Proteins and DNA are shown in green and orange, respectively. R = resistant line, GM = green monster parents. **a** Fur1 in complex with 5-FUMP. *Sc*Fur1 homology model, with a bound 5-FUMP analog (uridine monophosphate) taken from an aligned *holo Tm*Fur1 crystal structure (PDB ID: 1O5O). **b** Cdc60 model. Cdc60 homology model bound to a docked tavaborole molecule. **c** Top1 model. DNA-Top1-camptothecin complex, modelled using a *Sc*Top1 crystal structure (PDB: 1OIS), with bound camptothecin taken from an aligned *holo Hs*Top1p crystal structure (PDB: 1T8I). **d** Tor2 model. mTOR-rapamycin-Fpr1 tertiary complex model, modelled using a crystal structure of the human complex as a template (PDB ID: 4DRI^107^. mTOR residues 1001–2474 are shown in green (homology model), and Fpr1 is shown in yellow (crystal structure, PDB: 1YAT). **e** Fpr1-rapamycin model. Fpr-rapamycin (crystal structure, PDB 1YAT). **f** Tub2-nocodazol model. Tub2-nocodazol (crystal structure, PDB: 5CA1). **g** Act1 model. Act1 (crystal structure, PDB: 1YAG), bound to a docked hectochlorin molecule. Evaluation of hepatocellular traversal by *P. berghei* sporozoites using an established flow cytometry-based assay^108^. **h** Liver cell invasion assay. Flow cytometry plots show traversal and invasion of host cells at 2 h post-invasion by exoerythrocytic forms in Huh7.5.1 cells. The percent of rhodamine-dextran positive single cells (RD) was used to determine overall traversal frequency, controlled against cytochalasin D (positive) at 10 µM and infected untreated conditions, while invasion was evaluated by exclusive GFP + signal.

For compounds with known targets, we frequently identified mutations clustering in the active site of the proposed target molecule. For example, we isolated six clones resistant to flucytosine (Table 1, Supplementary Data 4). Of the nine identified missense or nonsense mutations, six were in the uracil phosphoribosyltransferase domain of *FUR1* (probability of enrichment by chance = 1.2 × 10^−26^ using a hypergeometric mean function). Resistance to 5-flucytosine has been reported in multiple clinical isolates of *Candida* and is typically caused by mutations in genes that encode the enzymes involved in the metabolic transformation of the prodrug. In a study of flucytosine-resistant clinical isolates of *C. albicans*, mutations in *Fur1* were identified^42^. A homology model (Fig. 4a) revealed that the amino acid changes identified in our in vitro selections are all located near the 5-FUMP binding pocket, suggesting that these changes confer resistance by disrupting 5-FUMP binding.

**Table 1:** Summary of statistically enriched genes identified in compound selections.

Gene	Description	NG	NC	Compounds	p-value
YRM1	Zn2-Cys6 zinc-finger transcription factor	52	13	See Supplementary Data 4	3.53 × 10^−116^
YRR1	Zn2-Cys6 zinc-finger transcription factor	48	12	See Supplementary Data 4	2.51 × 10^−105^
PMA1	Plasma membrane P2-type H + -ATPase	15	5	GNF-Pf-445, Hygromycin B, KAE609, Wortmannin, GNF-Pf-3891	3.00 × 10^−24^
BUL1	Ubiquitin-binding component of the Rsp5p E3-ubiquitin ligase complex	14	11	See Supplementary Data 4	3.92 × 10^−22^
PDE2	High-affinity cyclic AMP phosphodiesterase	13	2	MMV000570, MMV007181	4.76 × 10^−20^
TPO1	Polyamine transporter of the major facilitator superfamily	12	5	GNF-Pf-4283, MMV006389, CBR410, CBR572, TCMDC-124263	5.34 × 10^−18^
ANY1	Putative protein of unknown function	11	5	Amitriptyline, MMV019017, Clomipramine, MMV396736, Sertraline	5.51 × 10^−16^
BAP2	High-affinity leucine permease	10	6	GNF-Pf-3703, GNF-Pf-3815, GNF-Pf-5129, GNF-Pf-5468, MMV006389	5.19 × 10^−14^
SIP3	Putative sterol transfer protein	8	3	GNF-Pf-445, Lomerizine, Loratidine	3.38 × 10^−10^
INP53	Polyphosphatidylinositol phosphatase	8	1	MMV000442	3.38 × 10^−10^
AFT1	Transcription factor involved in iron utilization	7	3	MMV085203, MMV1007245, CBR868	2.28 × 10^−8^
PDR1	Transcription factor that regulates the pleiotropic drug response	7	7	DDD01027481, Doxorubicin, MMV000442, MMV007224, MMV667491, CBR668, CBR110	2.28 × 10^−8^
ERG9	Farnesyl-diphosphate farnesyl transferase	7	2	AN7973, MMV1078458	2.28 × 10^−8^
YAP1	Basic leucine zipper (bZIP) transcription factor	6	4	Cycloheximide, GNF-Pf-4739, DDD01027481, MMV001246	1.34 × 10^−6^
TOP2	Topoisomerase II	6	1	Etoposide	1.34 × 10^−6^
HXT3	Low-affinity glucose transporter of the major facilitator superfamily	6	3	Amitriptyline, DDD01035522, GNF-Pf-445	1.34 × 10^−6^
ERG11	Lanosterol 14-alpha-demethylase	6	2	MMV001239, CBR499	1.34 × 10^−6^
FUR1	Uracil phosphoribosyltransferase	6	1	Flucytosine	1.34 × 10^−6^
CCR4	Component of the CCR4-NOT transcriptional complex	5	4	GNF-Pf-2823, GNF-Pf-4583, MMV403679, CBR868	6.76 × 10^−5^
ERG3	C-5 sterol desaturase	5	2	Miconazole, Posaconazole	6.76 × 10^−5^
FKS1	Catalytic subunit of 1,3-beta-D-glucan synthase	5	4	DDD01027481, CBR113, CBR668, CBR110	6.76 × 10^−5^
CDC60	Cytosolic leucyl-tRNA synthetase	5	2	CBR668, Tavaborole	
ROX1	Heme-dependent repressor of hypoxic genes;	5	3	Loratadine, MMV665909, TCMDC-124263	6.76 × 10^−5^
PDR3	Transcriptional activator of the pleiotropic drug-resistance network	5	3	Lapatinib, MMV665794, CBR110	6.76 × 10^−5^
OSH3	Member of an oxysterol-binding protein family	5	1	Posaconazole	6.76 × 10^−5^
CSG2	Endoplasmic reticulum membrane protein	4	2	GNF-Pf-1618, KAAA726	4.76 × 10^−2^
ELO2	Fatty acid elongase	4	2	Doxorubicin, MMV667491	4.76 × 10^−2^
TUP1	General repressor of transcription	4	1	Diethylstilbestrol	
RPO21	RNA polymerase II largest subunit B220	4	4	Lapatinib, MMV007181, MMV1469689, CBR110	4.76 × 10^−2^
SUR2	Sphinganine C4-hydroxylase	4	1	MMV667491	4.76 × 10^−2^
VMA16	Subunit c” of the vacuolar ATPase	4	4	Lapatinib, MMV019017, MMV396736, MMV665882	4.76 × 10^−2^
PAN1	Part of actin cytoskeleton-regulatory complex Pan1p-Sla1p-End3p	4	2	Hygromycin B, KAE609	4.76 × 10^−2^

32 genes contained at least four independently selected coding mutations, the significance threshold for number of mutations occurring in a gene at a rate not expected by chance across the dataset. Bonferroni-corrected *p*-values were calculated using the hypergeometric mean function (number of successes in sample = number of times gene was identified as mutated; sample size = total number of genes mutated in dataset (731); successes in population = number of independent selections (355); population size = number of genes in yeast genome multiplied by 355) followed by Bonferroni-correction using number of independent selections.For a complete list of genes and mutations identified across the study, refer to Supplementary Data 4.

*NG* number of times gene was identified as mutated in independent evolution experiments, *NC* number of compounds.

We obtained four benzoxaborole-resistant clones using the antifungal drug, tavaborole. They were highly resistant and contained six SNVs, four of which (R316T, V400F, V400D, and M493R) were mutations in the 145 amino acid aminoacyl-tRNA synthetase editing domain of *CDC60* (*p* = 1.11 × 10^−18^, hypergeometric mean function), the gene that encodes leucyl-tRNA-synthetase in yeast. Wild-type yeast can evolve benzoxaborole resistance via amino acid changes both within the ligase editing site of the leucyl-tRNA synthetase and outside the active site^43–45^, indicating the relevance of the GM model. A LeuRS homology model (Fig. 4b) with a tavaborole ligand docked using QuickVina2^46^ suggests that the observed *CDC60* mutations could confer resistance by directly interfering with tavaborole binding to Cdc60.

We also examined compounds used in chemotherapy. Camptothecin is a specific topoisomerase (Top1) inhibitor that binds the DNA/Top1 cleavage complex, preventing DNA religation^47^. We isolated two camptothecin-resistant yeast clones with three missense mutations, two of which were in *TOP1* (G297C and E669*) (Supplementary Data 4). *TOP1* mutations can lead to camptothecin resistance in the human HCT116 colon adenocarcinoma cell line when exposed to SN38, a water-soluble camptothecin derivative^48,49^. A homology model (Fig. 4c)^50^ was constructed by aligning a partial yeast Top1 crystal structure to a crystal structure of human TOP1 with camptothecin bound (PDB: 1T8I)^51^. This model showed that G297 is located in the core domain of the enzyme near the binding pocket, suggesting that it confers drug resistance by directly impeding compound binding. E669* truncates the entire C-terminal domain, which contains the DNA-binding site^52^ (Fig. 4c), thus eliminating many protein/DNA contacts and likely impeding the formation of the drug-DNA-protein complex.

Rapamycin, a macrocyclic lactone, and its analog everolimus potently inhibit mTOR, a protein kinase component of both the mTORC1 and mTORC2 complexes that controls cell growth and proliferation in many species. Two rapamycin-resistant and three everolimus-resistant clones were isolated in our study. One carried a S1975I mutation in the FKBP12-rapamycin binding domain of mTOR, *TOR2*, and three carried a mutation in the FKBP-type peptidylprolyl cis-trans isomerase Pfam domain of FPR1, a small peptidylprolyl isomerase that interacts with mTOR (Supplementary Data 4). Recently, a selection with rapamycin in a *pdr1* deletion strain of *S. cerevisiae* BY4741^53^ identified mutations in TOR1, TOR2, and FPR1 that confer resistance to rapamycin^53^. One of the reported TOR2 mutations produced a S1975R amino acid change, the same residue we identified as being mutated in our study. A model of the yeast Tor2/Fpr1/rapamycin tertiary complex shows that residue S1975 is near the bound rapamycin molecule (Fig. 4d), suggesting that changes at this location might disrupt the formation of the tertiary complex. The model suggests that the two *FPR1* truncation mutations (Y33* and Q61fs) (Supplementary Data 4, Fig. 4e) likely confer resistance by interfering with everolimus binding.

Our collection also contained compounds active against other pathogens. For example, mebendazole, a benzimidazole compound, is among the few effective drugs available for treating soil-transmitted helminths (worms) in both humans and animals. It binds to tubulin, thereby disrupting worm motility^54^. We confirmed the antifungal activity^55^ of mebendazole and obtained two independent resistant clones with nine missense mutations, two of which were in the GTPase domain of the *TUB2* gene (R241S and L250F) (Supplementary Data 4), near or at the same residues (R241H and R241C) that confer resistance to the related antimitotic drug, benomyl, which also binds tubulin^53,56^. Modeling studies (Fig. 4f) confirm that the binding mode is similar to that of benomyl, which binds with high affinity to the beta subunit of tubulin, thereby disrupting the structure and function of microtubules^57^. Despite sharing a common target with yeast, helminths and nematodes have benzimidazole-resistance mutations in codons 167, 198, and 200, suggesting conservation of structure and function across phyla.

Alkylphosphocholines such as miltefosine and edelfosine were originally developed as anticancer agents, but recent work has shown that they are also effective against trypanosomatid parasites such as *Leishmania* and *Trypanosoma*^58–61^. The specific target of these drugs remains uncertain. Compound uptake in yeast is known to depend on the membrane transporter Lem3^62,63^, which facilitates phospholipid translocation by interacting with the flippase Dnf1^64^, and *DNF1* is closely related to the gene associated with miltefosine resistance in *Leishmania* (Ldmt (AY321297), BLASTP e = 2 × 10^−125^). We identified two independent *LEM3* mutations that confer resistance to miltefosine (K134* and Y107*) (Supplementary Data 4). Editing Y107* into the drug-naïve parent reconfirmed resistance to both miltefosine and edelfosine (Supplementary Data 7). Both mutations truncate the protein, functionally mimicking a deletion strain. *LEM3* is a yeast ortholog of LdROS (ABB05176.1, BLASTP 5 × 10^−13^), also related to *Leishmania* miltefosine resistance^65^.

### Revealing the putative target for an uncharacterized antimalarial natural product

Hectochlorin is a natural product from the marine cyanobacterium *Lyngbya majuscul*e^66^ that has strong antimalarial blood stage activity (IC_50_ = 85.60 nM ± 0.96) as well as activity against the ABC_16_-Green Monster strain (IC_50_ = 0.25 µM). We identified six independent disruptive mutations, three of which were in the actin Pfam domain of Act1 (*p* = 1.9 × 10^−13^, Supplementary Data 4). We confirmed by CRISPR-Cas9 editing that a mutation in *ACT1* confers resistance to hectochlorin in yeast (Supplementary Data 7). When mapped onto a crystal structure of Act1 (PDB: 1YAG^67^), the altered amino acids line a distinct protein pocket (Fig. 4g), suggesting they confer resistance by directly disrupting compound binding. To assess whether hectochlorin resistance in *Plasmodium* might occur through a similar mechanism, we also mapped the mutations onto a synthetic construct of *P. berghei* Act1 protein (PDB: 4CBW), which shares 97% sequence identity with *Pf*Act1. The altered amino acids again line a well-defined protein pocket, and the hectochlorin docked pose is also similar. This work supports published experiments that suggest actin is the target of hectochlorin^68^. To provide further support for this hypothesis, we determined if hectochlorin produces the same cell-invasion inhibition phenotype in malaria liver stage parasites as cytochalasin D, another actin polymerization inhibitor, which reduces *Plasmodium* sporozoite motility^69^. Treatment with 1 µM hectochlorin blocked parasite invasion as efficiently as 10 µM cytochalasin D (Fig. 4h).

### Mutations in the transcription factors YRR1 and YRM1 are associated with multidrug resistance in the ABC_16_-Green Monster yeast

**Fig. 5:**
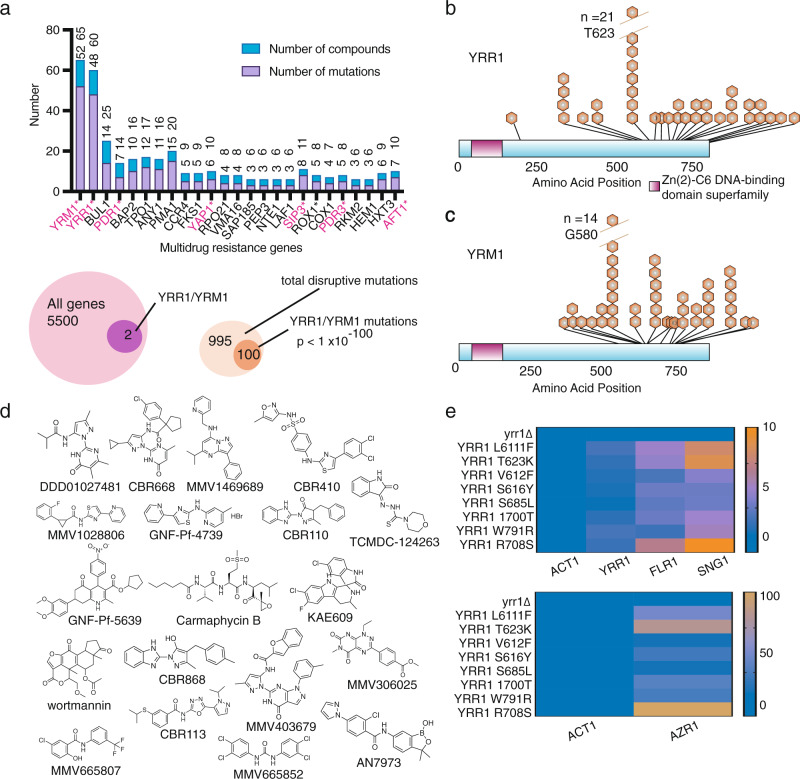
Mutations in transcription factors are over-represented. **a** Genes identified with three or more different compounds. Genes (8) with ontology GO:0140110 (transcription regulator activity) are shown in red. *YRR1* (**b**) and *YRM1* (**c**) mutation localization. Distribution of mutations in *YRR1* and *YRM1* across the amino acid sequence clustered in the C-terminal activation domain. **d** Scaffolds. Compounds used in selections resulting in *YRR1* and *YRM1* mutations. **e** *YRR1* single-nucleotide mutations but not loss-of-function mutations constitutively activate transcriptional targets. RT-qPCR was utilized to monitor mRNA levels of *YRR1* and *YRR1* activated genes. Clones with *YRR1* point mutations show increases in mRNA levels of *YRR1, SNG1, FLG1*, and *AZR1* relative to the wild-type strain (GM), but the deletion mutant does not. In the absence of *YRR1*, associated genes display baseline or lower level of expression. The heatmap indicates fold change normalized to *ACT1*.

SNVs in some genes appeared repeatedly across different compound sets. The set of 25 highest confidence genes (mutated five or more times across the dataset) was enriched for DNA-binding transcription factor activity (seven genes, Holm-Bonferroni-corrected *p* = 0.035). Altogether we observed 140 coding mutations in 24 genes affecting transcription (Fig. 5a). The greatest number of unique allelic exchanges was found in the two transcription factors, *YRR1* (27x) and *YRM1* (23x) (Fig. 5b, c). In addition, multiple unique missense mutations were observed in *PDR1* (7x), *PDR3* (5x) *YAP1* (5x), *AFT1* (5x), *TUP1* (3x) *HAL9* (2x), *AZF1* (2x) (Table 1, Supplementary Data 4). With the exception of *TUP1*, *AFT1*, and *YAP1*, all of these transcription factors contain the Zn_2_C_6_ fungal-type DNA-binding domain. In fact, we discovered 124 different mutations in 15 different Zn_2_C_6_ transcription factors. This Zn_2_C_6_ domain family (Pf00172) is only found in fungi: *S. cerevisiae* has 52 genes with this domain and *C. albicans* has 225. In this human pathogen, members include *FCR1, MRR2, TAC1, PDR1*, and *PDR3*, all genes involved in drug resistance. *YRR1 (PDR2), YRM1, PDR1,* and *PDR3* are all known to be involved in the pleiotropic drug response in *S. cerevisiae*^70,71^, activating transcription of drug transporters such as *PDR5, PDR10, PDR15, YOR1,* and *SNQ2* (reviewed in ref. ^72^). Although the GM strain has some of these multidrug efflux pumps deleted, the GM genome retains many other genes that could contribute to multidrug resistance that are under transcriptional control of *YRR1*^70^. In addition to statistical enrichment strongly suggesting a driving role in resistance, reverse gene editing by CRISPR/Cas9 of some of the mutations identified in *YRR1*, *AFT1*, and *TUP1* fully recapitulated the observed resistance phenotype (Supplementary Data 7).

Because the two Zn_2_C_6_ transcription factors *YRR1* and *YRM1* were mutated 100 times for 19 structurally diverse compounds (Fig. 5b–d), we conducted a focused investigation of the mutations and their spatial localization. Remarkably, all resistance-conferring *YRR1* and *YRM1* mutations were clustered in a ~170 amino acid domain in the C-terminal half of the protein (Fig. 5b, c), which is distal to the DNA-binding domain. We found no mutations in the DNA-binding domain and no mutations in the predicted activation domain at the far C-terminus. Notably, it has been shown that the C-terminal activation domain of Gal4 (activation domain 9aaTAD (857–871 aa), which interacts with Tra1 protein of the SAGA complex, can be substituted for the activation domain of Yrr1^70^. Yrr1 binds to the sequence (T/A)CCG(C/T)(G/T)(G/T)(A/T)(A/T), found upstream of genes involved in multidrug resistance such as *AZR1, FLR1, SNG1, SNQ2, APD1*, and *PLB1*^70,73,74^.

*YRR1* and *YRM1* are nonessential genes, and we hypothesized that our evolved resistant strains possessed *YRR1* and *YRM1* gain-of-function mutations that result in constitutive expression of transcriptional target genes encoding proteins with roles in drug resistance. This hypothesis is motivated in part by the fact that others have reported multidrug resistance-conferring gain-of-function mutations in the related genes *PDR1* and *PDR3*^75–77^. To expand on this previous work, we exposed a *YRR1* L611F strain (generated using CRISPR/Cas9) to a set of compounds and observed cross-resistance to almost all compounds tested (Supplementary Fig. 2, Supplementary Data 7). Others have found that deleting the *YRR1* gene entirely does not significantly increase sensitivity to cytotoxic compounds^75^, providing further evidence that the *YRR1* and *YRM1* mutations identified in our selections—which were all single amino acid changes—represent gain-of-function mutations. We confirmed this finding by testing a small set of cytotoxic compounds against a *yrr1* deletion strain^30^. With very few exceptions, we also noted no significant differences in growth inhibition over the wild-type-allele strain. Only compounds MMV6685852, GNF-PF-4739, and MMV668507 had different IC_50_s against the *yrr1* deletion strain and the fold IC_50_ differences were very modest.

To further test the hypothesis that the *YRM1* and *YRR1* SNVs are gain-of-function mutations resulting in constitutive expression of additional transporters and other potential resistance genes (including *AZR1, FLR1 SNG1, YLR046L, YLL046C, YPL088W*, and *YLR179C*^70^), we used qPCR to directly evaluate the expression of three such target genes (*AZR1*, *FLR1*, and *SNG1*). To this set we added the *YRR1* gene itself, since *YRR1* activates its own expression via an auto-feedback loop^76^ (Fig. 5e). We examined gene expression in *YRR1*-mutant strains, in the ABC_16_-Green Monster strain with a wild-type *YRR1* allele, and in the *yrr1* deletion clone (Supplementary Data 8). Relative RNA expression levels of the putative target genes *AZR1*, *FLR1, SNG1*, and *YRR1* are 1.5–140-fold higher in all *YRR1* evolved mutant clones tested compared to the parental GM strain; mutant clones also display elevated levels relative to the *yrr1* deletion clone (Fig. 5e). This data are further corroborated when using *TDH3* or *TAF10* as alternative housekeeping genes to *ACT1* (Supplementary Data 8).

To assess whether *YRR1* mutations confer resistance to specific compounds or elicit a more general resistance response, we evaluated seven different *YRR1*-mutant clones derived from our resistance selections for resistance to a set of five structurally unrelated compounds. All tested cultures showed strong cross-resistance to all tested compounds (Supplementary Data 7). Taken together, these results strongly support our hypothesis that the identified *YRR1* SNVs lead to constitutive transcriptional activation of its target genes involved in the pleiotropic drug response, thereby conferring general resistance to many compounds.

## Discussion

To our knowledge, this is one of the most comprehensive and systematic studies of the drug-selected mutational landscape in a model system like yeast. Although genome-wide sets of knockout strains have been used to discover drug targets and to study drug resistance^12,78–83^, our approach is different in that we identify SNVs in specific domains that might play a crucial role in compound-target interaction. Since these mutations confer resistance, we hypothesize that many of the SNVs are gain-of-function mutations. This difference in approach allows IVIEWGA to complement other genome-wide knockdown approaches, including those that rely on measures of haploinsufficiency in the presence of compound (HIPHOP)^78^. Few genetic tools are needed, and the approach can be applied to any organism that can be subjected to drug pressure and sequencing.

One potential disadvantage of whole-genome evolutionary approaches is that background passenger mutations can accumulate during the prolonged culturing of a fast-dividing organism and some may not contribute to resistance^40^. However, given the enrichment for nonsynonymous mutations in our study, most mutations likely do offer some advantage to the cell even when they are not the primary drivers of resistance. A large dataset like ours can provide clarity and statistical confidence regarding an allele’s importance even in the absence of CRISPR-Cas9 reconfirmation. The examples that are discussed in the manuscript did, in almost all cases, achieve strong statistical significance, with mutations in the same genes or with compounds that are structurally closely related appearing at rates much greater than expected by chance. It should be mentioned that many other mutations were found at rates not expected by chance and were not discussed here for the sake of brevity. For example, we found a strong association between *BUL1* and *BAP2* mutations and inhibitors of mitochondrial function as well as vacuolar ATPase mutations that were associated with other scaffold families.

The reproducibility of the results for genes and compound families indicates that the approach could be powerful in other fungal species that lack the genetic tools available for *S. cerevisiae*. Although others have identified targets of antifungal compounds through deep sequencing of drug-resistant mutants in other fungi^84^, *S. cerevisiae* is an excellent model for studying antifungal drug resistance in part because it is a haploid. IVIEWGA may be more difficult to apply in diploid species such as *Candida albicans* since complete loss-of-function mutations may be difficult to evolve and heterozygous gain-of-function mutations may be hard to identify in whole-genome sequencing data.

Another notable finding is the enrichment for Zn_2_C_6_ transcription factors *YRR1* and *YRM1* in the set of resistance genes. Though this may be specific to *Saccharomyces* or even the ABC_16_-Green Monster strain that was used, smaller-scale studies with specific compounds strongly support our findings (reviewed in ref. ^85^). Evolution experiments with unmodified *S. cerevisiae* and fluconazole gave mutations in *PDR1*^86^. Gallagher et al. mapped the ability of wild-type yeast to resist 4-nitroquinoline 1-oxide to *YRR1*^87^. In addition, *YRR1* was originally identified with selections in unmodified yeast^88^. Zn_2_C_6_ transcription factors are known to play a major role in the pathogenesis and pleiotropic drug response of pathogenic fungi such as *Candida* spp., the most common clinically relevant fungal pathogens^89–91^. Examples include *TAC1*, *STB5*, and many others (reviewed in ref. ^92^). Nevertheless, it is plausible that the abundance of *YRR1* and *YRM1* mutations that we identified only emerges when multiple other drug pumps are deleted, as in the GM strain.

A major unanswered question is how the cell senses increased compound pressure and translate this to increased transcription. A cofactor could bind to the drug-resistance domain in Yrr1 and Yrm1. Alternatively, the observed amino acid changes could increase either the strength of the dimerization of Yrr1 and/or its binding to DNA, causing an increase in the transcription of drug pumps, which would lead to resistance to many compounds irrespective of their MOA. This hypothesis is supported by the fact that (1) the RNA expression levels of the Yrr1 target genes *AZR1*, *FLR1, SNG1*, and *YRR1* are 2–70-fold higher in all of the tested *YRR1-*mutant clones when compared to the parental GM strain or the *yrr1* deletion lineage and (2) clones with *YRR1* mutations confer resistance even to compounds that were not used in selections or that did not yield *YRR1* mutations.

The World Health Organization has declared antimicrobial resistance one of the top 10 public health concerns currently facing humanity. While understanding resistance profiles and the rate at which resistance emerges are already key components of drug development pipelines for eukaryotic pathogens like the human malaria parasite *Plasmodium falciparum*^93^, studies of drug resistance in fungal pathogens remain extremely limited. Adaptive laboratory evolution has proven a useful tool for cataloging resistance variants across multiple microbes, enabling strategic drug design efforts and more. If the goal is to create better drugs for fungal pathogens, more studies like these are urgently needed.

## Materials and methods

### Yeast strains and clones

All yeast strains and clones used are listed in Supplementary Data 10.

### Statistics and reproducibility

To calculate probabilities of enrichment chance for specific allele sets a high accuracy hypergeometric mean function (Excel) was used:

$$P(X \, \ge \, x)=\frac{\left(\begin{array}{c}{k}\\ {x}\end{array}\right)\left(\begin{array}{c}{N-k}\\ {n-x}\end{array}\right)}{\left(\begin{array}{c}{N}\\ {n}\end{array}\right)}$$

n is the number of different observed alleles in a particular domain or gene for a compound in n selections.

x is the number of independent selections that were performed for a compound.

N is the number of nucleotides in the gene or domain of interest (e.g., 300 bases), or the number of genes in the genome multiplied by the number of n selections.

K is the number of nucleotides in the yeast genome (12.1 Mb), or the number of genes (6000) multiplied by the number of n selections.

A Bonferroni adjustment using the yeast genome size (12.1 × 10^6^) or a number of genes (6000) was made to correct for multiple hypothesis testing.

### *S. cerevisiae* susceptibility and dose-response assays

To measure compound activity against whole-cell yeast, single colonies of the ABC_16_-Green Monster strain were inoculated into 2 mL of YPD media and cultured overnight at 250RPM in a shaking incubator at 30 °C. Cultures were diluted the following day and 200 µl of log-phase cultures, (OD_600_nm readings between 0.1 and 0.2) were added to the wells of a 96-well plate. Eight 1:2 serial dilutions of the compound were subsequently performed, in biological duplicates, with final compound concentrations ranging from 0.1 to 150 μM. After an initial reading of OD_600_ (time 0 h), the plate was placed in an incubator at 30 °C for 18 h, and OD_600_ nm determined. IC_50_ values were calculated by subtracting OD_600_ nm values at time 0 h from time 18 h. Nonlinear regression on log([inhibitor]) vs. response with variable slope was performed using GraphPad Prism. As a negative control, cultures not treated with any compounds were run in parallel.

### GM Growth inhibition evaluation for the MMV Malaria Box, Pathogen Box, and Charles River libraries

96-well plates containing 10 µl 10 mM compounds were provided. We first tested these compounds for cytotoxicity of the ABC_16_-Green Monster clone in single-dose measurements (150 µM, in biological duplicates). Compounds that inhibited GM growth by at least 70% after 18 h (in either replicate) were further characterized using an eight-point dose-response assay (Supplementary Data 1).

### In vitro resistance evolution

In vitro selections^26–30^ were performed as outlined in Fig. 2. At least five independent selections were initiated with a single colony of the ABC_16_-Green Monster strain. Assorted drug concentrations (corresponding to 1-fold to 3-fold IC_50_) of diverse compounds were added to 50 mL conical tubes containing 50 μL of saturated (OD_600_ = 1.0–1.5; 1–5 × 10^7^/ml cells) ABC_16_-Monster cells in 20 mL of YPD medium. The tubes were then cultured at 250RPM in a shaking incubator at 30 °C. Cultures that achieved OD_600_ values between OD_600_ = 1.0–1.5; ~14 generations) were diluted 1:400 into fresh media with the inhibitors, and multiple rounds of dilutions (about of 2–6 rounds of dilutions) were performed at increasingly higher concentrations. After a culture was able to grow at a compound concentration that was at least equal to 2–3× IC_50_ compared to the parental IC_50_, cells from this polyclonal culture were plated onto agar plates containing the inhibitor. Single colonies were isolated, and an eight-point dose-response assay (in biological duplicates) with two-fold dilutions steps and final compound concentrations ranging from 0.1 to 150 µM (depending on the original parental IC_50_) was performed to determine the IC_50_ values of the evolved versus parental clones. Genomic DNA from clones that had at least an IC_50_ shift of 1.5-fold was extracted using the YeaStar Genomic DNA kit (cat. No D2002, ZYMO Research).

### Whole-genome sequencing and analysis

Sequencing libraries using 200 ng genomic DNA were prepared using the Illumina Nextera XT kit (Cat. No FC-131-1024, Illumina) following the standard dual indexing protocol, and sequenced on the Illumina HiSeq 2500 in RapidRun mode to generate paired-end reads at least 100 bp in length. Reads from the paired-end FASTQ files were aligned to the *S. cerevisiae* 288 C reference genome (assembly R64-1-1) using BWA-mem and further processed using Picard Tools (http://broadinstitute.github.io/picard/). Quality control, alignment, and preprocessing workflows were automated using the computational platform Omics Pipe^94^ to ensure scalable and parallelized analysis. A total of 363 clones were sequenced to an average coverage of 54.6× with an average of 99.7% reads mapping to the reference genome. Additional sequencing quality statistics are given in Supplementary Data 3. SNVs and INDELs were first called against the 288 C reference genome using GATK HaplotypeCaller, filtered based on GATK recommendations^95^, and annotated with a SnpEff^96^ database built from the *S. cerevisiae* 288 C (assembly R64-1-1) GFF to leave only high-quality variants with high allelic depth. Variants that were present in both the drug-sensitive parent clone and resistant clones were then subtracted using a custom shell script so that mutations were only retained if they arose during the compound selection process. To standardize and streamline the analysis, only one of the eight parent clones was used for the subtraction of variants (NODRUG--GM2). However, all 7 parent clones were analyzed for their own background mutations against NODRUG--GM2 first so that any background mutations could be removed following the first subtraction against NODRUG--GM2.

In total, 1405 mutations (1286 SNVs and 119 INDELs) met these criteria and were collated into a single list for subsequent analyses (Supplementary Data 4). Mutations were visually inspected using the Integrative Genomics Viewer (IGV)^97^. Manual annotation of variants was required in some cases to resolve issues with SnpEff outputs. Raw sequencing data files were uploaded to NCBI Sequence Read Archive under accession PRJNA590203. To increase the depth of our analysis, we also reanalyzed FASTQ files from several resistance selections that were previously published (https://escholarship.org/uc/item/42b8231t) and deposited in the NCBI Short Read Archive with the following accession numbers: SRX1745463, SRX1745464, SRX1745465, SRX1745466, SRX1751863, SRX1751950, SRX1751953, SRX1751954, SRX1805319, SRX1805320, SRX1805321, SRX1805322, SRX1805323, SRX1868845, SRX1869272, SRX1869274, SRX1869275, SRX1869276, SRX1869277, SRX1869278, SRX1869279, SRX1869280, SRX1869282). These include selections with the following compounds: KAE609, MMV001239, cycloheximide, MMV000570, MMV007181, MMV019017, and MMV396736.

### CNV analysis

Read coverage values across defined gene intervals in each alignment file were calculated using GATK DiagnoseTargets (input parameters: -max 2000 -ins 1500 -MQ 50). Coverage values were log-transformed then mean-centered across and within arrays in Cluster (http://bonsai.ims.u-tokyo.ac.jp/~mdehoon/software/cluster). Copy number variants were filtered so that they would only be retained if there was at least 2–3× fold coverage change relative to the parent strain and if they spanned four or more genes (Supplementary Data 5). CNVs were visually confirmed in IGV.

### Intergenic mutation analysis

A Python script was written to map the 271 intergenic mutations to the coordinates of known chromosomal features based on the GFF supplied on SGD for the *S. cerevisiae* S288C genome version R64-2-1. For each intergenic mutation position, we determined whether it was located within a feature and/or if it was located within 500 bp either upstream or downstream of a known coding gene. All possible annotations for each intergenic mutation position are listed in Supplementary Data 6.

### CRISPR-Cas9 allelic exchange in *S. cerevisiae*

CRISPR-Cas9 genome engineering was performed using the *S. cerevisiae* ABC_16_-Green Monster strain^29^. gRNA plasmids were generated with specific oligonucleotides (Supplementary Data 9) for the desired allelic exchange (Integrated DNA Technologies) containing a 24 bp overlap with the p426 vector backbone. Subsequently, target-specific gRNAs were PCR amplified/transformed into competent *E. coli* cells and selected on LB-Ampicillin plates. ABC_16_-Monster cells expressing Cas9 were simultaneously transformed with 300–500 ng of gene-specific gRNA vector and 1–2 nmole of synthesized donor template (IDT) via a standard lithium acetate method. Transformed cells were plated and selected on methionine and leucine deficient CM-glucose plates. Each engineered mutation was confirmed by Sanger sequencing (Eton Bioscience).

### qPCR

*S. cerevisiae* strains were grown in YPD (1% yeast extract, 2% bacto peptone, 2% dextrose) overnight at 30^o^C. 1 OD_600_ log-phase cells were harvested and subject to total RNA extraction using Qiagen RNeasy kit, following the manufacturer’s protocol. cDNA was generated using ThermoFisher SuperScript IV First-Strand Synthesis System, following the manufacturer’s protocol using oligo(dT). qPCR was performed with oligonucleotides (Supplementary Table 1) in technical triplicate with Quanta PerfeCTa SYBR® Green FastMix. Analysis was done using Prism 8. Ct values for each gene of interest were averaged and normalized against *ACT1* within each clone (ΔCt). Then each gene of interest was normalized against corresponding genes in the wild-type GM background (ΔΔCt). Fold expression was calculated using the formula: 2^-ΔΔCt^^98^. This analysis was done for each of the four biological replicates (Supplementary Data 8).

### Plasmodium invasion assay

The impact of hectochlorin on hepatocellular traversal and invasion by *Plasmodium berghei* (Pb) sporozoites was measured using a flow cytometry-based assay^69^. *Anopheles stephensi* mosquitoes infected with GFP expressing Pb sporozoites (Pb-GFP)^99^ were purchased from the New York University (NYU) Insectary Core Facility. Approximately 24 h before infection, 1.75 × 10^5^ Huh7.5.1 cells were seeded in 24-well plates using DMEM (Invitrogen cat# 11965-092) supplemented with 10% FBS (Corning cat# 35-011-CV) and 1× Pen Strep Glutamine (100 Units/mL Penicillin, 100 µg/mL Streptomycin, and 0.292 mg/mL L-glutamine) (Invitrogen cat# 10378-016) for a final volume of 1 mL. On the day of infection, hectochlorin was added to test wells (final concentration 1 µM) with cytochalasin D (final concentration 10 µM) acting as a positive control for invasion inhibition. A non-infected control and DMSO (final concentration 0.5%) negative control was also utilized to mimic the treated well conditions. Sporozoites were freshly dissected and prepared 2–4 h before infection^100^. Immediately prior to infection, rhodamine-dextran was added to each test well (final concentration 1 mg/mL) followed by 3.5 × 10^4^ Pb-GFP sporozoites. The plates were then incubated at 37 °C and 5% CO_2_ for 2 h. Following this incubation, the cells were washed and the presence of GFP and rhodamine-dextran signals were evaluated using flow cytometry.

### Model building

We used I-TASSER^50^ to model proteins without acceptable structures in the Protein Data Bank^101^. To visually inspect the homology models, we aligned them to the structural templates used for model construction^102^. We discarded models that had poor I-TASSER C-scores or that we judged to be improbable (e.g., excessively disordered). Where there were no homologous crystal structures with bound ligands for reference, we used docking to predict ligand binding poses. Specifically, we converted the SMILES strings of the ligands to 3D structures using a beta version of Gypsum-DL^103^ and docked the 3D models using QuickVina2^46^ (exhaustiveness = 15). The AutoDock forcefield does not include parameters for boron. To dock tavaborole, we substituted the boron atom with a carbon atom, as recommended on the AutoDock webpage (http://autodock.scripps.edu). We tested both the “C” and “A” atom types as boron substitutes to determine which gave predicted tavaborole poses with the best binding affinities. For heme groups, we manually added a charge of +2 to the iron atom. All protein-structure images were generated using BlendMol^104^.

### Reporting summary

Further information on research design is available in the Nature Research Reporting Summary linked to this article.

## Supplementary information

Supplementary Materials

Description of Additional Supplementary Files

Supplementary Data 1

Supplementary Data 2

Supplementary Data 3

Supplementary Data 4

Supplementary Data 5

Supplementary Data 6

Supplementary Data 7

Supplementary Data 8

Supplementary Data 9

Supplementary Data 10

Reporting Summary

## Data Availability

Raw DNA sequences for all 363 yeast clones have been deposited in the Sequence Read Archive (www.ncbi.nlm.nih.gov/sra) under BioProject accession PRJNA590203. All other data are available in the manuscript or the supplementary materials. This work is licensed under a Creative Commons Attribution 4.0 International (CC BY 4.0) license, which permits unrestricted use, distribution, and reproduction in any medium, provided the original work is properly cited. To view a copy of this license, visit http://creativecommons.org/licenses/by/4.0/. This license does not apply to figures/photos/artwork or other content included in the article that is credited to a third party; obtain authorization from the rights holder before using such material.
