# Supplementary material for: Adaptive laboratory evolution in S. cerevisiae highlights role of transcription factors in fungal xenobiotic resistance
Source: Commun Biol. 2022 Feb 11;5:128. doi: 10.1038/s42003-022-03076-7 (PMC8837787; doi:10.1038/s42003-022-03076-7)
Supplement: Supplementary file 1 — Supplementary Materials [file 42003_2022_3076_MOESM1_ESM.pdf]

SUPPLEMENTARY MATERIALS

Adaptive laboratory evolution in *S. cerevisiae* highlights the role of transcription factors in fungal xenobiotic resistance

Sabine Ottilie[1,†], Madeline R. Luth[1,†], Erich Hellemann[2,#], Gregory M. Goldgof[1,#], Eddy Vigil[1], Prianka Kumar[1], Andrea L. Cheung[1], Miranda Song[1], Karla P. Godinez-Macias[1], Krypton Carolino[1], Jennifer Yang[1], Gisel Lopez[1], Matthew Abraham[1], Maureen Tarsio[3], Emmanuelle LeBlanc[4], Luke Whitesell[4], Jake Schenken[1], Felicia Gunawan[1], Reysha Patel[1], Joshua Smith[1], Melissa S. Love[5], Roy M. Williams[1,11], Case W. McNamara[5], William H. Gerwick[6], Trey Ideker[7], Yo Suzuki[8], Dyann F. Wirth[9,10], Amanda K. Lukens[10], Patricia M. Kane[3], Leah E. Cowen[4], Jacob D. Durrant[2], Elizabeth A. Winzeler[1*]

Affiliations:

[1]Department of Pediatrics, University of California, San Diego, Gilman Dr., La Jolla, CA 92093, USA

[2]Department of Biological Sciences, University of Pittsburgh, 4249 Fifth Avenue, Pittsburgh, PA, 15260, USA

[3]Department of Biochemistry and Molecular Biology, SUNY Upstate Medical University, Syracuse, New York 13210, USA

[4]Department of Molecular Genetics, University of Toronto, Toronto, ON, M5G 1M1, Canada

[5]Calibr, a division of The Scripps Research Institute, La Jolla, CA 92037, USA

[6]Center for Marine Biotechnology and Biomedicine, Scripps Institution of Oceanography, La Jolla, California, 92037, USA

[7]Department of Medicine, University of California San Diego, La Jolla, CA, USA

[8]Department of Synthetic Biology and Bioenergy, J. Craig Venter Institute, La Jolla, 92037 CA, USA

[9]Department of Immunology and Infectious Diseases, Harvard T.H. Chan School of Public Health, Boston, Massachusetts, USA

[10]Infectious Disease and Microbiome Program, Broad Institute, Cambridge, MA 02142, USA

[11]Present address: Aspen Neuroscience, San Diego, CA 92121, USA

[†]These authors contributed equally

[#]These authors contributed equally

*Correspondence: ewinzeler@health.ucsd.edu

CONTENTS

Supplementary Figure 1. Resistant clones IC_{50} Shift Fold Change.

Supplementary Figure 2. *YRR1* deletion and *YRR1* L611F mutants show different levels of drug resistance to a variety of chemotypes.

Supplementary Table 1. Oligos used in qPCR experiments.

SUPPLEMENTARY FIGURES

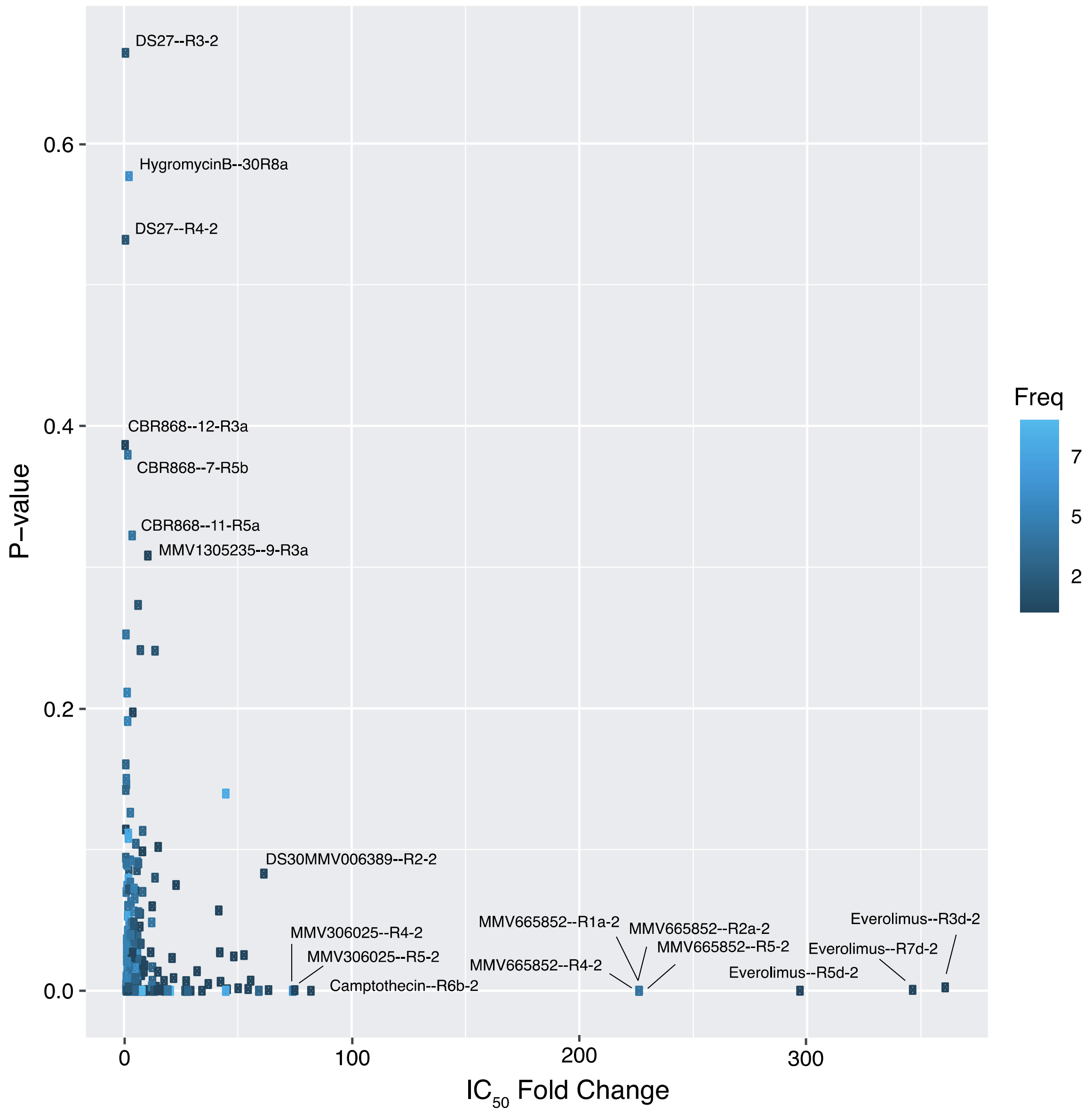

Supplementary Figure 1. Resistant clones IC50 Shift Fold Change. Scatter plot for fold change of resistant clones and their p-value as obtained from Supplementary Data 2. Plot shows the frequency of IC_{50} fold change as observed in the different resistant clones, in which lighter blue color indicates a high frequency and darker blue a lower frequency of fold change. 121 clones had a 1.5- to 5-fold change, 101 clones a 5- to 1-fold and 98 clones had more than 10-fold change. The graphic was generated using R 3.61 and ggplot2 library.

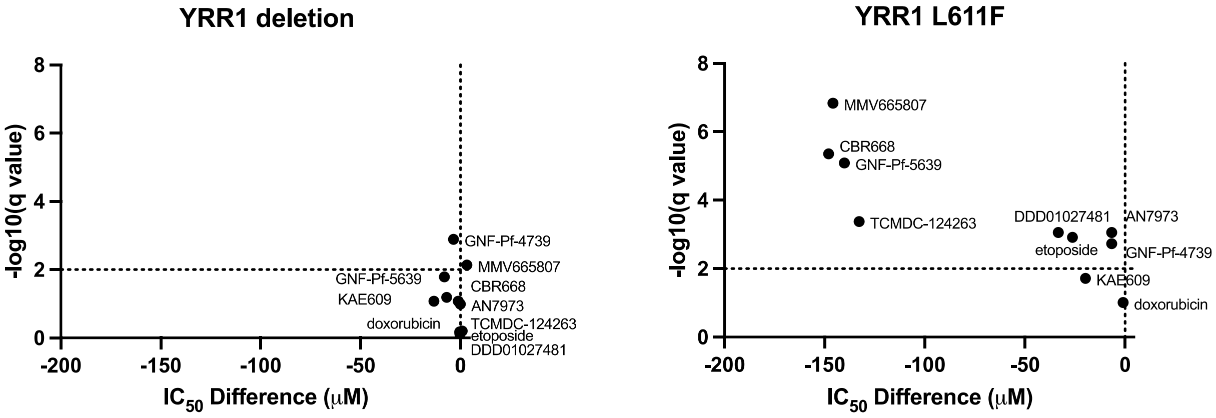

Supplementary Figure 2. *YRR1* deletion and *YRR1* L611F mutants show different levels of drug resistance to a variety of chemotypes. Values are the IC_{50} difference (calculated as described in Methods) relative to the ABC_{16}-Green Monster parent and the log10(q value) of observing the difference by chance using multiple unpaired t-tests. Compounds were tested in biological triplicates. Raw values are provided in Supplementary Data 7.

SUPPLEMENTARY TABLE

Supplementary Table 1. Oligos used in qPCR experiments. Gene expression of the genes listed here were monitored in the various *YRR1* mutant clones of *S. cerevisiae*, as these SNVs in *YRR1* correlate to upregulation of associated genes, as rationale for the gain of function phenotype, the increased resistance to different compounds. Gene expression was monitored via RT-qPCR using SYBR green and the oligos presented in this table. The forward and reverse primers bind to the corresponding gene transcripts to generate dsDNA that is measured through dye intercalation.

Gene Name	Forward Primer	Reverse Primer
ACT1	CGTCTGGATTGGTGGTTCTATC	GGACCACTTTCGTCGTATTCTT
AZR1	CTCTGAGATCGGGTGGTTATTT	CATGGTCTCCTTGAATCCGATAG
FLR1	TAGGGTGCGTACTTGCTTATG	GACACACATTGCCACGATTAAA
SNG1	GGCAGAGGAGGATTCGTAGTAT	TCAGGTATGGAGGGCAGTAAG
YRR1	TCGCCAAATTTCCCTCCTTTA	CCGGTCGGCATATGGATTTAT
TDH	CGGTAGATACGCTGGTGAAGTTTC	TGGAAGATGGAGCAGTGATAACAAC
TAF-10	ATATTCCAGGATCAGGTCTTCCGTAGC	GTAGTCTTCTCATTCTGTTGATGTTGTTGTTG
