# Supplementary material for: Adaptive laboratory evolution in S. cerevisiae highlights role of transcription factors in fungal xenobiotic resistance
Source: Commun Biol. 2022 Feb 11;5:128. doi: 10.1038/s42003-022-03076-7 (PMC8837787; doi:10.1038/s42003-022-03076-7)
Supplement: Supplementary file 2 — Description of Additional Supplementary Files [file 42003_2022_3076_MOESM2_ESM.pdf]

Description of Additional Supplementary Files

File name: Supplementary Data 1
Description: Library description and enriched clusters.

File name: Supplementary Data 2
Description: IC50 values for each resistant clone and relative fold-shift compared to the affiliated parent clone.

File name: Supplementary Data 3
Description: Sequencing statistics for the parents and drug-resistant yeast clones generated for this study.

File name: Supplementary Data 4
Description: Aggregate list of the 1,405 high-quality mutations (1,286 SNVs and 119 INDELs) that arose over the course of compound selection in each of the 355 compound-resistant clones.

File name: Supplementary Data 5
Description: List of 24 Copy Number Variant (CNV) events observed in compound-resistant clones.

File name: Supplementary Data 6
Description: Annotations of the intergenic mutations identified in compound-resistant clones.

File name: Supplementary Data 7
Description: CRISPR/Cas9 confirmation IC50s.

File name: Supplementary Data 8
Description: RT-qPCR of YRR1 and associated genes in wild-type (GM) and mutant yeast strains conferring resistance.

File name: Supplementary Data 9
Description: Oligos used to knock in putative resistance-conferring mutations.

File name: Supplementary Data 10
Description: Yeast clone genotypes.
